# Understanding the long‐term impact of the COVID‐19 pandemic on non‐muscle‐invasive bladder cancer outcomes: 12‐Month follow‐up data from the international, prospective COVIDSurg Cancer study

**DOI:** 10.1002/bco2.432

**Published:** 2024-10-15

**Authors:** Cameron E. Alexander, Arjun Nathan, Alexander Light, Chuanyu Gao, Vinson Chan, Sinan Khadhouri, Kevin Gallagher, Kevin G. Byrnes, Michael Walters, Terry Hughes, Rita Perry, Kelvin Okoth, Laura Magill, Thomas Pinkney, Joseph B. John, John S. McGrath, Alexandra Colquhoun, Yuhao Zhang, James Blackmur, Eric Etchill, Stanley Tang, Damián García Escudero, Grant Stewart, Veeru Kasivisvanathan, COVIDSurg Collaborative, Shomik Sengupta, Christopher Ip, Joshua Kealey, Alison Blatt, Ahmad Alam, Natalie Lott, Ashrarur Rahman Mitul, Nazmul Islam, Sabbir Karim, Yuen‐Chun Jeremy Teoh, On‐Ting Erica Chan, Chi‐Fai Ng, Chi‐Hang Yee, Rajkumar Kottayasamy Seenivasagam, Gurpremjit Singh, Neha Mishra, Ankur Mittal, Vikas Kumar Panwar, Shanky M. K. Singh, Sanjeev Misra, Jeewan Ram Vishnoi, Puneet Pareek, Gaurav Aggarwal, Suvraraj Das, Sujoy Gupta, Gagan Prakash, Ganesh Bakshi, Uday Chandankhede, Mahendra Pal, Luca Morelli, Matteo Bianchini, Annalisa Comandatore, Gregorio Di Franco, Lorenzo Fatucchi, Niccolò Furbetta, Desirée Gianardi, Simone Guadagni, Matteo Palmeri, Alberto Porcu, Teresa Perra, Massimo Madonia, Alessandro Tedde, Matteo Tedde, Riccardo Schiavina, Matteo Droghetti, Lorenzo Bianchi, Crescenzo Cacciapuoti, Francesco Costa, Pierpaolo Bordoni, Francesco Fleres, Guglielmo Clarizia, Alessandro Spolini, Ildar Fakhradiyev, Tanabayeva Shynar, Saliev Timur, Andee Dzulkarnaen Zakaria, Jien Yen Soh, Aimatnuddin Husiari Hussain, Mohd Nizam Md Hashim, Mohamed Ashraf Mohamed Daud, Mohamad Fadli Mohd Yunus, Michael Pak‐Kai Wong, Rosnelifaizur Ramely, Wan Zainira Wan Zain, Zaidi Zakaria, Guillermo Feria‐Bernal, Gerardo Tena‐González Méndez, Ricardo A. Castillejos‐Molina, Fernando Gabilondo‐Navarro, Bernardo Gabilondo‐Pliego, Carlos E. Méndez‐Probst, Mariano Oropeza‐Aguilar, Francisco Rodríguez‐Covarrubias, Héctor Sandoval‐Barba, Mariano Sotomayor, Sosa Duran Erik Efrain, Ziad Aboharp Hasan, Alberto Bazan Soto, Abiodun Okunlola, Oluseyi Banjo, Peter Egharebva, Musliu Adetola Tolani, Oyelowo Nasir, Omolara Williams, Kazeem Atobatele, Olufunmilade Omisanjo, Selmy Awad, S. A. L. E. H. Alghamdi, Soliman Ghedan, Waleed Althobaiti, Uros Bumbasirevic, Zoran Dzamic, Boris Kajmakovic, Bogomir Milojevic, Marko Zivkovic, Victor Javier García Porcel, Jose David Jiménez Parra, Olimpia Molina Hernández, Julián Oñate Celdrán, Carlos Sánchez Rodríguez, José Gil‐Martínez, Felipe Alconchel, Tatiana Nicolás‐López, Vitharanage Srimantha Dewsiri Rodrigo, Umesh Jayarajah, Kavinda Deshapriya Bandara, Fanourios Georgiades, Islam Abu‐Nayla, Igor Chipurovski, Alberto Coscione, Bhavan Rai, Ashwin Sachdeva, Kamran Haq, Gianmarco Isgro, Clio Kennedy, Tobias Klatte, James Manners, Lyndon Gommersall, Megan Thomas, Mark Kitchen, Justine Royle, Jelizaveta Pereca, Gianluca Maresca, Holly Bekarma, Zulahdi N‐Aabulsi, Thomas Walton, Paul Lloyd, Feng Tse, Ben Eddy, Mark Yao, Issam Ahmed, Sashi Kommu, Georgios Papadopoulos, Adrian Simoes, Edward Streeter, Milan Thomas, Alexander Laird, Connor Boyle, Ian McAllister, Jennifer Foreman, Michael Ng, Nicholas Campain, John Pascoe, Pamela Murray, Omikunle Babawale, Joel Bowen, Wendy Enticott, Christina Fontaine, Naomi Neal, Matthew Byrne, Ibrahim Jour, Ganesh Sathanapally, James Catto, Steve Bromage, Zara Gall, Magda Kujawa, E. Charles Osterberg, Pooja Srikanth, Hannah Kay, Vishal Patel, Arjun Srivastava, Adan Tijerina, Alodia Gabre‐Kidan, Hillary Jenny, Benjamin Bigelow, Mitchell Ladd, Chao Long, Harsha Malapati, Sarah Rapaport, Lillian Tsai, Dominique Vervoort, Lekha Yesantharao, Chad Markey, Andrew Loehrer, Margaret Hanley, James Mc Andrew Jones, Chiamaka Lawrencia Okorie

**Affiliations:** ^1^ Luton and Dunstable University Hospital Luton UK; ^2^ British Urology Researchers in Surgical Training (BURST) London UK; ^3^ Division of Surgery and Interventional Sciences University College London London UK; ^4^ Department of Surgery and Cancer Imperial College London London UK; ^5^ William Harvey Hospital East Kent Hospitals University Foundation Trust Kent UK; ^6^ Leeds Institute of Medical Research University of Leeds Leeds UK; ^7^ School of Medicine University of St Andrews St Andrews United Kingdom; ^8^ Institute of Genetics and Cancer University of Edinburgh Edinburgh UK; ^9^ Birmingham Centre for Observational and Prospective Studies University of Birmingham Birmingham UK; ^10^ University of Exeter Medical School Exeter UK; ^11^ Royal Devon University Healthcare NHS Foundation Trust Exeter UK; ^12^ Cambridge University Hospitals NHS Foundation Trust Cambridge UK; ^13^ Stepping Hill Hospital Stockport NHS Foundation Trust Stockport UK; ^14^ Johns Hopkins Hospital Baltimore Maryland USA; ^15^ Hospital General Reina Sofía Murcia Spain; ^16^ Department of Surgery University of Cambridge Cambridge United Kingdom

**Keywords:** bladder cancer, COVID‐19, delay, non‐muscle invasive, pandemic, surgery

## Abstract

**Objective:**

The objective of this study was to report the 12‐month oncological outcomes for patients with non‐muscle‐invasive bladder cancer (NMIBC) within the prospective, international COVIDSurg Cancer study.

**Patients and methods:**

Eligible patients were aged ≥18 years and scheduled for elective surgical management of NMIBC with curative intent (transurethral resection of bladder tumour [TURBT] or bladder biopsy) from 21 January to 14 April 2020. The primary outcome was disease recurrence within 12 months of previous elective TURBT/bladder biopsy. Secondary outcomes included disease progression within 12 months of previous elective TURBT/bladder biopsy, site‐declared delay to surgery from diagnosis as a consequence of COVID‐19 and deviation in standard care due to COVID‐19. Comparisons were made to cohorts from the pre‐pandemic era.

**Results:**

Bladder cancer accounted for 2.2% (*n* = 446) of patients in the COVIDSurg Cancer study, with data contributed by 27 centres across 12 countries internationally. Within this included cohort, 229 patients had NMIBC and 12‐month follow‐up data available. On application of National Institute for Health and Care Excellence (NICE) criteria, 47.2% were classified as having high‐risk disease. Overall disease recurrence and progression rates were 29.3% and 9.7% at 12 months, respectively. In purely high‐risk pre‐pandemic cohorts, the International Bladder Cancer Group (IBCG) estimates a recurrence rate of 25% at 12 months, and the European Association of Urology (EAU) NMIBC 2021 scoring model estimates a 12‐month progression rate of 3.5%. As a consequence of the COVID‐19 pandemic, 10.9% of patients had site‐declared delay to TURBT/bladder biopsy; 7.4% did not undergo intravesical therapy or had early discontinuation of this; 9.2% did not undergo early repeat resection for high‐risk disease; and 18.3% had a delay to cystoscopic follow‐up surveillance.

**Conclusions:**

This prospective study indicates that there were widespread deviations in usual care for NMIBC during the pandemic and that 12‐month oncological outcomes appear to be impaired compared to published pre‐pandemic outcomes.

## 1 INTRODUCTION

The global pandemic caused by severe acute respiratory syndrome coronavirus 2 (SARS‐CoV‐2) and associated coronavirus disease 2019 (COVID‐19) led to profound disruption in the delivery of cancer services internationally.^1^, ^2^ In the context of urological malignancies, global survey data have demonstrated that delays were not limited to elective cancer surgery but impacted on timely referral and diagnostics, as well as the delivery of systemic treatment and follow‐up protocols.^3^ The high risk of serious complications from COVID‐19 seen in cancer patients, as well as those undergoing general anaesthesia, had particular implications for those with bladder cancer, who typically represent an elderly cohort with high levels of co‐morbidity.^4^, ^5^, ^6^ In the survey performed by Action Bladder Cancer UK during the first wave of COVID‐19, 49% of bladder cancer patients in the United Kingdom reported disruption to treatment or follow‐up.^7^

In order to balance the need for risk stratification and clinical prioritisation of services against the risk of COVID‐19 infection, there was a rapid accumulation of new clinical guidance from professional medical bodies, local government and international urological associations.^8^, ^9^ This extended to the management of patients with bladder cancer, but inevitably such recommendations were limited by the relative lack of evidence to inform an entirely unprecedented and rapidly changing global emergency.^10^, ^11^

The COVIDSurg Cancer study was an international, prospective cohort study that included over 20 000 patients and provided an analysis of the impact of COVID‐19 on planned cancer surgery for 15 cancer types across 61 countries.^12^ Non‐muscle invasive bladder cancer (NMIBC) represents a common but heterogenous disease that requires a high proportion of resources within urological services.^13^ There remains uncertainty regarding the extent of global deviations in NMIBC care that occurred during the COVID‐19 pandemic and, in particular, what the implications have been for oncological outcomes.

The objective of this study was to report the international 12‐month oncological outcomes for patients with NMIBC within the COVIDSurg Cancer study who were scheduled for elective surgery (transurethral resection of bladder tumour [TURBT]/bladder biopsy) during the COVID‐19 pandemic and to compare these with expected outcomes from robust, published sources in pre‐pandemic times.

## 2 PATIENTS AND METHODS

### 2.1 Study design and participants

The methodology used to identify eligible patients and hospital sites and perform data collection has been previously described by the COVIDSurg Cancer study.^12^ This was an international, multi‐centre, prospective cohort study. Patients were eligible for inclusion in the COVIDSurg Cancer study where they were aged ≥18 years and scheduled for elective surgical management of a solid cancer with curative intent. Eligible patients were identified over a 3‐month period from the time of local emergence of COVID‐19; this was defined locally by centres based on the date on which the first notification of SARS‐CoV‐2 was confirmed, ranging from 21 January to 14 April 2020. There were no restrictions on participants by geographical location, with all hospitals worldwide eligible to participate.

This study is a sub‐group analysis of patients who had a diagnosis of NMIBC, as defined by their post‐operative pathology and staging. This included patients with Ta or T1 disease, and carcinoma in situ (CIS) (in isolation or combination with Ta/T1 disease), and either Nx or N0, Mx or M0 disease (i.e., no clinical suspicion for nodal or metastatic disease).^14^ Patients with a new diagnosis of NMIBC (i.e., primary presentation) and those with recurrent disease were included. To account for international variations in the application of grading systems, users were able to select between high versus low grade, or G1 versus G2 versus G3 disease. The UK‐based National Institute for Health and Care Excellence (NICE) risk stratification system was applied in the categorisation of disease risk.^15^

### 2.2 Outcomes

Whilst the initial follow‐up for all cancers within the COVIDSurg Cancer study ceased on 31 August 2020, those sites that submitted data for urological cancers were contacted to submit 12‐month outcome data between 1 July 2021 and 10 January 2022. The primary outcome for this study was disease recurrence within 12 months of the date of previous elective surgery (i.e., TURBT or bladder tumour biopsy). The secondary outcomes included disease progression within 12 months of the date of previous elective TURBT/bladder biopsy (defined as progression to high‐risk disease or muscle invasive or nodal/metastatic disease); the presence of delay to surgery from cystoscopic diagnosis as a consequence of COVID‐19; or the presence of deviation in standard care (i.e., avoidance or early discontinuation of intravesical therapy; avoidance of early repeat cancer resection/biopsy; delay to cystoscopic surveillance) as a consequence of COVID‐19.

Given the inclusion of an international cohort, the associated heterogeneity between health‐care systems and cancer pathways, and a relative lack of consensus on the definition of ‘delay’ in NMIBC cancer care, this study did not enforce a fixed definition of COVID‐19‐related delay. Individual participating sites were required to make their own circumstantial judgements as to whether there had been a clear and direct deviation in the usual standard of care at their institution as a consequence of COVID‐19.

### 2.3 Data collection and statistical analysis

Approvals to conduct the study and/or ethical exemption, as required, were obtained by all participating sites. Study data were collected and managed using Research Electronic Data Capture (REDCap) tools hosted by at the University of Birmingham.^16^, ^17^ All statistical analyses were performed by author KO using Stata SE version 17.0. Data on patient characteristics were summarised using appropriate descriptive statistics; continuous variables were summarised using mean (standard deviation) for data with normal distribution or median (interquartile range) for data with skewed distribution, and categorical variables were summarised using numbers (%). Multivariable logistic regression models were fitted to examine independent factors associated with disease recurrence (dependent variable) at 12 months; independent factors, including COVID‐19 related delay to surgery, were selected based on previous literature and clinical knowledge, with a *p*‐value of <0.05 considered statistically significant. Validated scoring models developed in pre‐pandemic NMIBC cohorts to estimate the likelihood of disease recurrence and progression were identified to facilitate contextualisation of outcome data from this cohort; this included the 2006 European Organisation for Research and Treatment of Cancer (EORTC) scoring model (and subsequent application of EORTC data by the International Bladder Cancer Group), as well as the European Association of Urology (EAU) NMIBC 2021 scoring model.^18^, ^19^, ^20^

## 3 RESULTS

**FIGURE 1:**
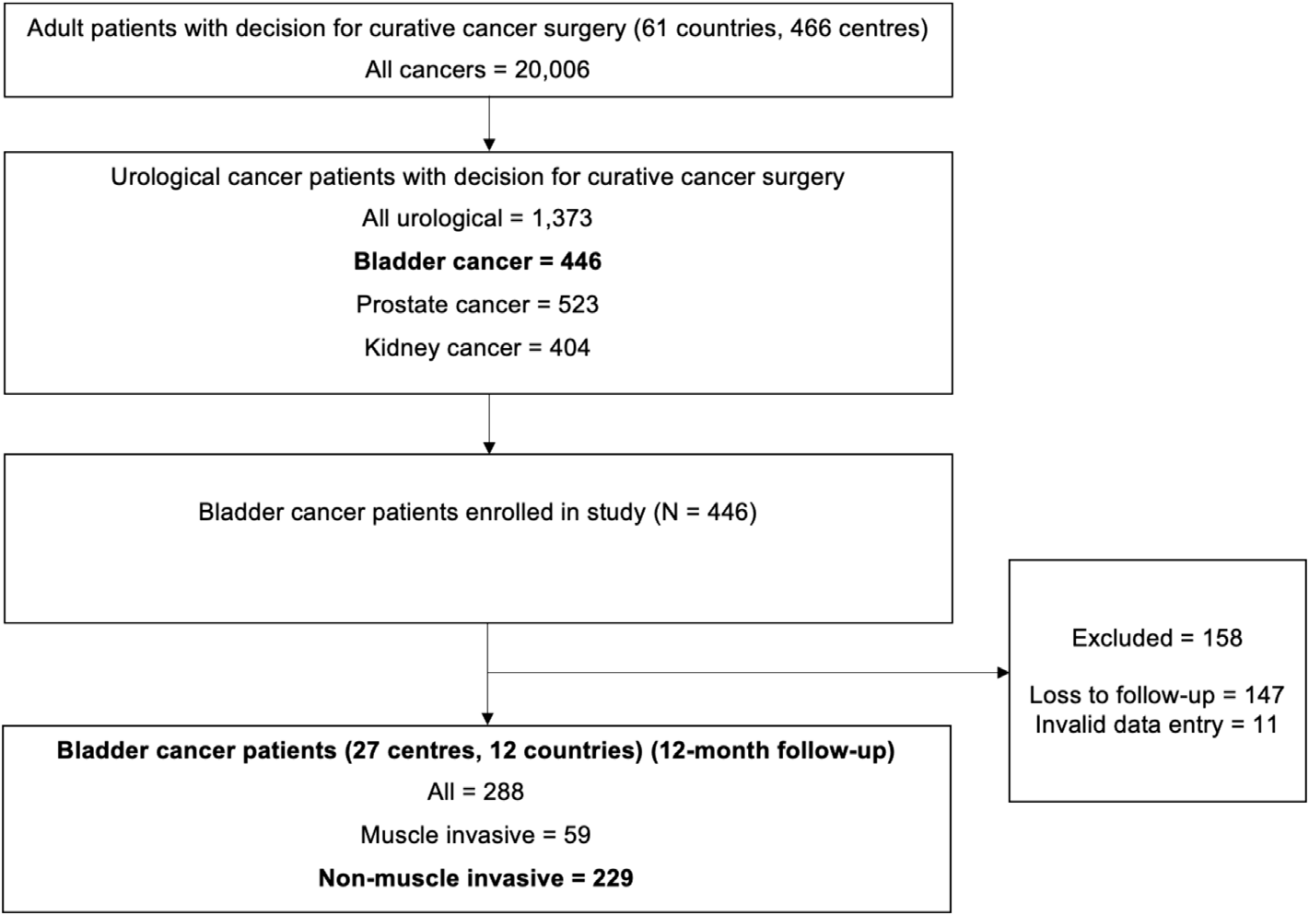
Flowchart of included patients.

Patients with a diagnosis of bladder cancer accounted for 2.2% (*n* = 446) of all patients included in the COVIDSurg Cancer study, with data contributed by 27 centres across 12 countries internationally. Follow‐up data were collected for 64.6% (*n* = 288) of all bladder cancer patients at 12 months, with the remaining patients lost to follow‐up (33.0%, *n* = 147) or excluded due to invalid data entry (2.4%, *n* = 11). Of the patients with 12‐month follow‐up data, 229 (79.5%) patients had NMIBC and were included in this study (Figure 1). The countries that contributed the largest proportion of patients to the included cohort were the United Kingdom (45.0%), Spain (12.7%), Australia (11.4%), India (10.9%), United States (8.7%) and China (7.9%).

The majority of patients were aged ≥60 years (83.4%) and male (79.0%), and 12.7% were current smokers (Table 1). The majority of patients had a new primary diagnosis of NMIBC (68.6%) rather than recurrent disease (31.4%), and according to NICE risk classification, 47.2% had high‐risk disease (Table 2).^15^

**TABLE 1:** Patient characteristics—non‐muscle invasive bladder cancer cohort.

Patient characteristic (*n* = 229)	Frequency, *n* (%)
Age, years	
<50	10 (4.4)
50–59	23 (10.0)
60–69	63 (27.5)
70–79	71 (31.0)
≥80	57 (24.9)
Sex	
Male	181 (79.0)
Female	48 (21.0)
BMI	
≥30	54 (23.5)
Current smoker	
Yes	29 (12.7)
ECOG performance status	
0	121 (52.8)
1	69 (30.1)
≥2	39 (17.0)

Abbreviation: ECOG, Eastern Cooperative Oncology Group.

**TABLE 2:** Disease characteristics—non‐muscle invasive bladder cancer cohort.

Disease characteristic (*n* = 229)	Frequency, *n* (%)
Status	
Primary diagnosis (first presentation)	157 (68.6)
Recurrent disease	72 (31.4)
Pathological stage	
CIS only	12 (5.2)
Ta	140 (61.1)
T1	77 (33.6)
Concurrent CIS	
Yes	31 (13.5)
Grade (Option 1) (*n* = 126)	
Low	60 (47.6)
High	66 (52.4)
Grade (Option 2) (*n* = 91)	
G1	5 (5.5)
G2	43 (47.3)
G3	43 (47.3)
NICE risk stratification^a^	
Low	65 (28.4)
Intermediate	54 (23.6)
High	108 (47.2)

Abbreviation: CIS, carcinoma in situ.

^a^ National Institute for Health and Care Excellence (NICE) (2015).^15^

A total of 10.9% (*n* = 25) of patients had a delay to surgery (TURBT or bladder biopsy) as a consequence of COVID‐19, with inadequate critical care, theatre or hospital bed capacity cited as the most frequent reasons for delaying surgery (9.2%, *n* = 21/229) (Table 3). The median time from diagnosis at cystoscopy to surgery in those patients with a COVID‐19‐related delay to surgery was 42 days (range 21 to 105), compared to 21 days (range 7 to 35) for those without COVID‐19 related delay.

**TABLE 3:** Deviations in non‐muscle invasive bladder cancer care due to COVID‐19.

COVID‐19‐related deviations in care (*n* = 229)	Frequency, *n* (%)
Delay to surgery (TURBT or bladder biopsy)	
Yes	25 (10.9)
Factors contributing to delayed surgery^a^ (*n* = 25)	
Patient tested positive for COVID‐19	1 (0.4)
Patient choice to delay surgery	5 (2.2)
MDT decision to delay surgery	6 (2.6)
Inadequate critical care, theatre or hospital bed capacity	21 (9.2)
Professional society guideline recommendations	1 (0.4)
Avoidance of intravesical therapy	
Yes (all)	11 (4.8)
Single post‐operative installation of mitomycin C avoided	5 (2.2)
Adjuvant course of Bacillus Calmette‐Guerin (BCG) avoided	6 (2.6)
Early discontinuation of intravesical therapy course	
Yes (all)	6 (2.6)
Adjuvant course of mitomycin (early discontinuation)	1 (0.4)
Adjuvant course of BCG (early discontinuation)	5 (2.2)
Avoidance of early repeat TURBT or bladder biopsy (high risk disease)	
Yes	21 (9.2)
Delay to cystoscopic surveillance (follow‐up)	
Yes	42 (18.3)

Abbreviations: MDT, multidisciplinary team; TURBT, transurethral resection of bladder tumour.

^a^ Participants allowed to select all (i.e., multiple) contributing factors that were applicable.

As a consequence of COVID‐19, 7.4% (*n* = 17/229) of patients did not undergo intravesical therapy or had early discontinuation of this. Within this group, a single post‐surgical installation of mitomycin C was entirely avoided in 2.2% (*n* = 5/229), and an adjuvant course of mitomycin was discontinued early in 0.4% (*n* = 1/229); an adjuvant course of Bacillus Calmette‐Guerin (BCG) was entirely avoided in 2.6% (*n* = 6/229) and discontinued early in 2.2% (*n* = 5/229) (Table 3). Furthermore, 9.2% (*n* = 21/229) did not undergo early repeat resection for high‐risk disease, and 18.3% (*n* = 42/229) had a delay to cystoscopic follow‐up surveillance as a consequence of COVID‐19 (Table 3).

The overall disease recurrence and progression rates for the included cohort were 29.3% and 9.7% at 12 months, respectively. In contrast to this, and in exclusively high‐risk, pre‐pandemic NMIBC cohorts, the IBCG has previously estimated a recurrence rate of 25% based on EORTC data at 12 months, and the EAU NMIBC 2021 scoring model has estimated a progression rate of 3.5% at 12 months (Figure 3; Figure 4).^18^, ^19^, ^20^

**FIGURE 2:**
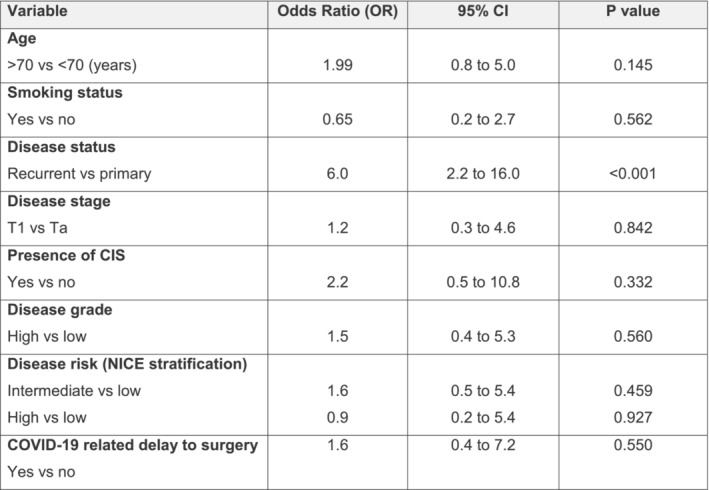
Multivariate model assessing the independent association between COVID‐19 related delay to surgery and disease recurrence at 12 months. CIS, carcinoma in situ; NICE, National Institute for Health and Care Excellence.

The recurrence rate at 12 months for patients with site‐declared COVID‐19‐related delay to surgery was 28.0% (*n* = 7/25) compared to 29.4% (*n* = 60/204) for those without COVID‐19‐related surgical delay. On multivariable analysis, there was no independent association between COVID‐19‐related delay to surgery and recurrence at 12 months (odds ratio [OR] 1.6; 95% CI 0.4 to 7.2; *p* = 0.550) (Figure 2).

## 4 DISCUSSION

This study assessed the 12‐month oncological outcomes of patients with NMIBC included within the prospective, international, multi‐centre COVIDSurg Cancer study.^12^ As such, it included NMIBC patients planned for elective surgery with curative intent during the first global wave of COVID‐19 and provides insights into the major deviations in care that faced urological health‐care services globally in this period.

The application of the UK NICE risk classification system for NMIBC used in this study does provide challenges in undertaking a direct comparison in 12‐month cancer outcomes between this cohort and those where other risk classification systems have been applied.^15^ Furthermore, comparisons between this cohort and other studies need to take account of the inclusion of mixed risk groups in this study (28% low risk; 24% intermediate risk; 47% high risk according to NICE).

**FIGURE 3:**
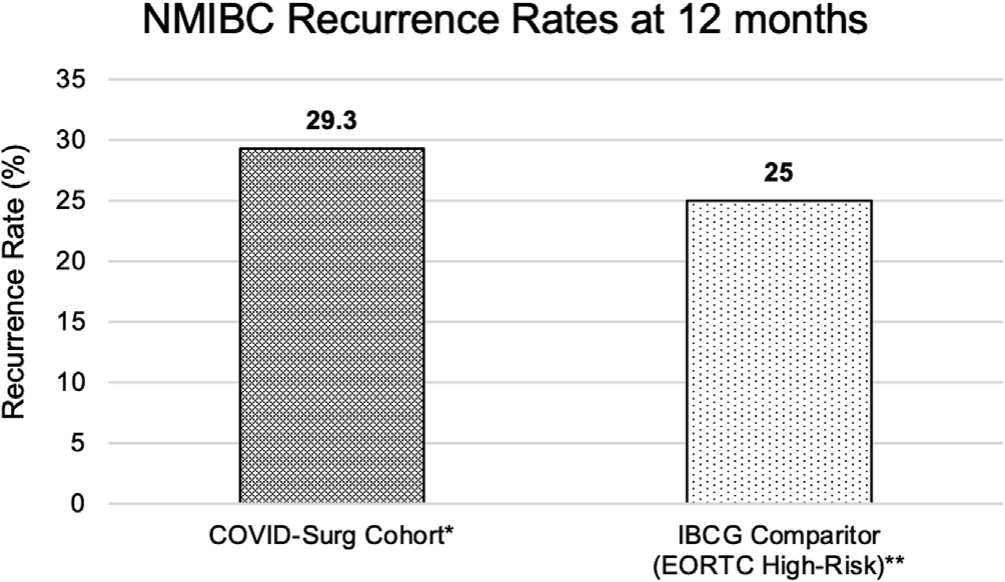
Comparison of 12‐month recurrence rates between COVID‐Surg NMIBC cohort and International Bladder Cancer Group High‐Risk (2006 EORTC scoring model). *COVID‐Surg NMIBC cohort applied UK National Institute for Health and Care Excellence (NICE) risk stratification with this cohort composed of 47% high‐risk, 24% intermediate‐risk and 28% low‐risk disease patients. **The International Bladder Cancer Group (IBCG) estimated the recurrence rate at 12 months using European Organisation for Research and Treatment of Cancer (EORTC) data for those with high‐risk disease, defined as those with histologically confirmed T1 and/or high‐grade disease and/or carcinoma in situ (CIS) for patients that had not yet undergone Bacillus Calmette‐Guerin (BCG).^18^

**FIGURE 4:**
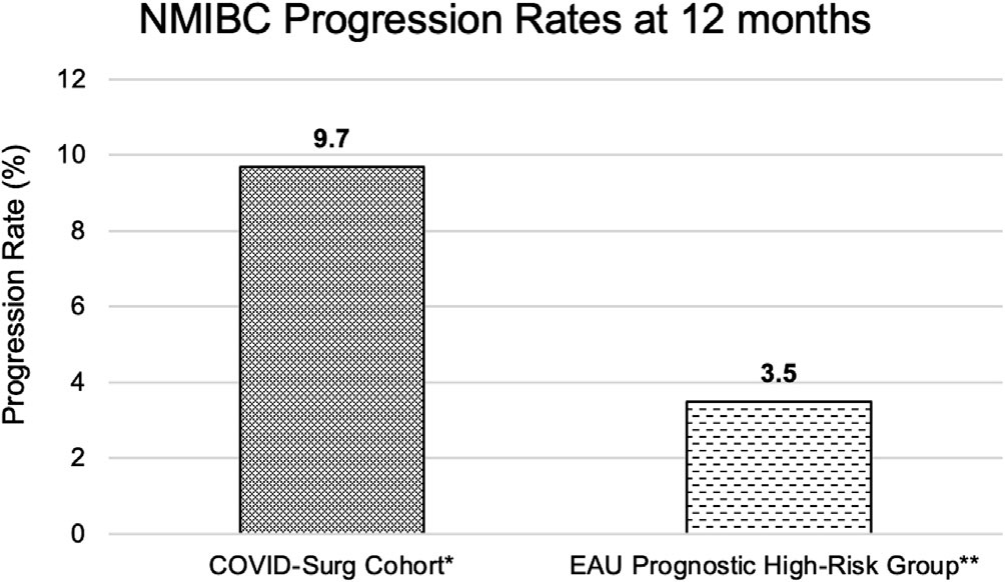
Comparison of 12‐month progression rates between COVID‐Surg NMIBC cohort and EAU Prognostic High‐Risk Group (2021 EAU NMIBC scoring model). *COVID‐Surg NMIBC cohort applied UK National Institute for Health and Care Excellence (NICE) risk stratification with this cohort composed of 47% high risk, 24% intermediate risk and 28% low risk. **The European Association of Urology developed a scoring model to calculate NMIBC disease progression, with high risk defined as all T1/HG/G3 disease without carcinoma in situ (CIS) except those in very high‐risk group; all CIS patients except those in very high‐risk group; and the inclusion of other stages/grades where there is the presence of additional risk factors (2021 EAU NMIBC scoring model).^20^

Despite this, indirect comparisons appear to suggest that 12‐month NMIBC outcomes have been compromised in this COVID cohort. Whilst the recurrence rate at 12 months for this mixed cohort was 29.3% (only 47% classified as NICE high‐risk disease), the IBCG has previously estimated a recurrence rate of 25% at 12 months for those with purely high‐risk disease based on the adoption of data from the EORTC scoring model (Figure 3).^18^, ^19^ Furthermore, whilst the rate of disease progression in this cohort was 9.7% at 12 months, the EAU NMIBC 2021 scoring model reports a 12‐month progression rate of 3.5% for those with high‐risk disease (Figure 4) (16% for very high‐risk).^20^

The impact of COVID‐19‐related delay to surgery on recurrence was a particular focus of this study, but no clear difference was observed between those with delay (median 42 days from diagnosis to surgery, recurrence 28.0% at 12 months) and those without recorded delay (median 21 days, recurrence 29.4% at 12 months). It is possible that the 1‐year follow‐up remains too short to demonstrate a clear difference between these relatively small groups in terms of recurrence and that such differences will become apparent over time. Indeed, in Sylvester and colleagues' combined analysis from seven EORTC trials, the median time to recurrence was 2.7 years.^19^ Meaningful statistical comparisons between such sub‐groups here are, however, limited by the relatively small cohort in which delay to surgery was reported (*n* = 25).

Tulchiner and colleagues demonstrated that NMIBC patients presenting during the COVID‐19 pandemic (2020) were significantly more likely to present with T1 disease (19.1% vs. 6.8%) or with high‐grade disease (63.8% vs. 37.9%) when compared with pre‐pandemic levels (2019).^21^ In this cohort, the upper limit for those experiencing COVID‐19‐related delay in diagnosis to TURBT/bladder biopsy was 105 days, and Wallace and colleagues have previously reported a significant difference in survival at 1, 3 and 5 years beyond a surgical wait threshold of 68 days for urothelial cancer.^22^ Although this study has not demonstrated a clear detrimental impact of COVID‐related delay to surgery on 12‐month recurrence rates, additional analyses of the included cohort do suggest that patients with delay were more likely to have T1 disease (56.0% vs. 31.2%), G3 disease (36.0% vs. 16.8%) or NICE‐classified high‐risk disease (80% vs. 43.6%), compared to those without delay. The potential for other confounding variables to have influenced these observations means that it is not possible to draw definitive conclusions on causation; in particular, the potential co‐existence of delay to diagnosis in health‐care systems where there was also delay to treatment cannot been accounted for here.

It is clear that there were also other international deviations from the typical standard of care for NMIBC during the first wave of the COVID‐19 pandemic, including the avoidance of intravesical therapy or early re‐resection and delay to cystoscopic surveillance. The clinician‐reported levels of disruption reported in this study may represent relatively conservative estimates when compared to other studies; in the survey performed by Action Bladder Cancer UK, 53% of patients reported postponement or cancellation of cystoscopy, and 70% reported delay or postponement of intravesical therapy.^7^ An important likely source of sampling bias within this study is that those centres that were well‐resourced or the least affected by the pandemic would likely have been more able to participate in the study.

Whilst EAU guidance did recommend that TURBT should be undertaken within 6 weeks for an established bladder lesion and haematuria, it is also suggested that re‐resection could be deferred by 6 months (if visibly complete resection and T1 disease with muscle) or that cystoscopic surveillance and intravesical therapy could be deferred by 6 months for intermediate‐risk NMIBC.^9^, ^11^ Given that the overall oncological outcomes of this cohort appear to have been impaired compared to pre‐pandemic cohorts, this suggests that any future de‐escalations of care within existing pathways for NMIBC should be undertaken with caution and careful assessment of impact.

It is important to recognise that not all factors that may have influenced outcomes have been recorded within the study and that ultimately cancer outcomes are reliant on complex, multi‐faceted pathways that vary by individual country. This study did not capture, for example, delays in referral or presentation to secondary care; reports of UK practice in April 2020 suggested that suspected cancer referrals by primary care physicians had reduced by 60% compared to the previous 3 years.^23^ It would seem likely that delay to diagnosis would have been a co‐existing issue in those institutions and health‐care systems experiencing delay to surgery. Whilst this study provides a unique insight into the collective deviations of care and cancer outcomes of an international cohort, the majority of data were contributed by high‐income health systems and do not take account of the heterogeneity across international health‐care systems. The loss to follow‐up, relatively small cohort size and 12‐month outcome period limited the ability of this study to identify predictors of poorer outcomes in adequately powered multivariable models.

## 5 CONCLUSION

This prospective study indicates that there were widespread deviations in usual care for NMIBC during the pandemic and that 12‐month oncological outcomes appear to be impaired compared to published pre‐pandemic outcomes. This study highlights the importance of high‐quality care for NMIBC outcomes as well as cautioning any future de‐escalations of care within existing pathways for NMIBC.

## AUTHOR CONTRIBUTIONS

Study oversight was delivered by Veeru Kasivisvanathan and Grant Stewart. Michael Walters, Terry Hughes, Rita Perry, Laura Magill and Thomas Pinkney were responsible for data collection and management. Data analysis was performed by Kelvin Okoth. All authors were responsible for data interpretation, manuscript development and review.

## CONFLICT OF INTEREST STATEMENT

All authors declare no conflict of interest.

## Supporting information


**Appendix S1** COVIDSurg Collaborative authors.
