# Supplementary material for: Understanding the long‐term impact of the COVID‐19 pandemic on non‐muscle‐invasive bladder cancer outcomes: 12‐Month follow‐up data from the international, prospective COVIDSurg Cancer study
Source: BJUI Compass. 2024 Oct 15;5(11):1044–51. doi: 10.1002/bco2.432 (PMC11557269; doi:10.1002/bco2.432)
Supplement: Supplementary file 1 — Appendix S1 COVIDSurg Collaborative authors. [file BCO2-5-1044-s001.docx]

**Appendix 1 – COVIDSurg Collaborative Authors**

Shomik Sengupta, Christopher Ip, Joshua Kealey, Alison Blatt, Ahmad Alam, Natalie Lott, Ashrarur Rahman Mitul, Nazmul Islam, Sabbir Karim, Yuen-Chun Jeremy Teoh, On-Ting Erica Chan, Chi-Fai Ng,

Chi-Hang Yee, Rajkumar Kottayasamy Seenivasagam, Gurpremjit Singh, Neha Mishra, Ankur Mittal, Vikas kumar Panwar, Shanky MK Singh, Sanjeev Misra, Jeewan Ram Vishnoi, Puneet Pareek, Gaurav Aggarwal,

Suvraraj Das, Sujoy Gupta, Gagan Prakash, Ganesh Bakshi, Uday Chandankhede, Mahendra Pal, Luca Morelli, Matteo Bianchini, Annalisa Comandatore, Gregorio Di Franco, Lorenzo Fatucchi, Niccolò Furbetta,

Desirée Gianardi, Simone Guadagni, Matteo Palmeri, Alberto Porcu, Teresa Perra, Massimo Madonia,

Alessandro Tedde, Matteo Tedde, Riccardo Schiavina, Matteo Droghetti, Lorenzo Bianchi, Crescenzo Cacciapuoti, Francesco Costa, Pierpaolo Bordoni, Francesco Fleres, Guglielmo Clarizia, Alessandro Spolini, Ildar Fakhradiyev, Tanabayeva Shynar, Saliev Timur, Andee Dzulkarnaen Zakaria, Jien Yen Soh, Aimatnuddin Husiari Hussain, Mohd Nizam Md Hashim, Mohamed Ashraf Mohamed Daud, Mohamad Fadli Mohd Yunus, Michael Pak-Kai Wong, Rosnelifaizur Ramely, Wan Zainira Wan Zain, Zaidi Zakaria, Guillermo Feria-Bernal, Gerardo Tena-González Méndez, Ricardo A Castillejos-Molina, Fernando Gabilondo-Navarro, Bernardo Gabilondo-Pliego, Carlos E Méndez-Probst, Mariano Oropeza-Aguilar, Francisco Rodríguez-Covarrubias, Héctor Sandoval-Barba, Mariano Sotomayor, Sosa Duran Erik Efrain,

Ziad Aboharp Hasan, Alberto Bazan Soto, Abiodun Okunlola, Oluseyi Banjo, Peter Egharebva, Musliu Adetola Tolani, Oyelowo Nasir, Omolara Williams, Kazeem Atobatele, Olufunmilade Omisanjo, Selmy Awad, SALEH ALGHAMDI, Soliman Ghedan, Waleed Althobaiti, Uros Bumbasirevic, Zoran Dzamic, Boris Kajmakovic, Bogomir Milojevic, Marko Zivkovic, Victor Javier García Porcel, Jose David Jiménez Parra, Olimpia Molina Hernández, Julián Oñate Celdrán, Carlos Sánchez Rodríguez, José Gil-Martínez, Felipe Alconchel, Tatiana Nicolás-López, VITHARANAGE SRIMANTHA DEWSIRI RODRIGO, Umesh Jayarajah, Kavinda Deshapriya Bandara, Fanourios Georgiades, Islam Abu-Nayla, Igor Chipurovski, Alberto Coscione, Bhavan Rai, Ashwin Sachdeva, Kamran Haq, Gianmarco Isgro, Clio Kennedy, Tobias Klatte, James Manners, Lyndon Gommersall, Megan Thomas, Mark Kitchen, Justine Royle, Jelizaveta Pereca, Gianluca Maresca, Holly Bekarma, Zulahdi A-Nabulsi, Thomas Walton, Paul Lloyd, Feng Tse, Ben Eddy, Mark Yao, Issam Ahmed, Sashi Kommu, Georgios Papadopoulos, Adrian Simoes, Edward Streeter, Milan Thomas, Alexander Laird, Connor Boyle, Ian McAllister, Jennifer Foreman, Michael Ng,

Nicholas Campain, John Pascoe, Pamela Murray, Omikunle Babawale, Joel Bowen, Wendy Enticott,

Christina Fontaine, Naomi Neal, Matthew Byrne, Ibrahim Jour, Ganesh Sathanapally, James Catto, Steve Bromage, Zara Gall, Magda Kujawa, E Charles Osterberg, Pooja Srikanth, Hannah Kay, Vishal Patel, Arjun Srivastava, Adan Tijerina, Alodia Gabre-Kidan, Hillary Jenny, Benjamin Bigelow, Mitchell Ladd, Chao Long, Harsha Malapati, Sarah Rapaport, Lillian Tsai, Dominique Vervoort, Lekha Yesantharao, Chad Markey, Andrew Loehrer, Margaret Hanley, James McAndrew Jones, Chiamaka Lawrencia Okorie
